# Cohort Profile: The Dale-Wonsho health and demographic surveillance system, Southern Ethiopia

**DOI:** 10.1093/ije/dyae018

**Published:** 2024-02-09

**Authors:** Alemu Tamiso Debiso, Kebede Tefera, Netsanet Abera Asseffa, Yilkal Simachew, Frehiwot Atsibeha, Fanuel Belayneh, Beekam Kebede Olkeba, Tihun Feleke, Gezahegn Bekele, Solomon Asnake, Selamawit Mengesha, Mesay Hailu, Andargachew Kassa

**Affiliations:** College of Medicine and Health Sciences, Hawassa University, Hawassa, Ethiopia; College of Medicine and Health Sciences, Hawassa University, Hawassa, Ethiopia; College of Medicine and Health Sciences, Hawassa University, Hawassa, Ethiopia; College of Medicine and Health Sciences, Hawassa University, Hawassa, Ethiopia; College of Medicine and Health Sciences, Hawassa University, Hawassa, Ethiopia; Dale-Wonsho Health Demographic Surveillance Site, Yirgalem, Ethiopia; College of Medicine and Health Sciences, Hawassa University, Hawassa, Ethiopia; College of Medicine and Health Sciences, Hawassa University, Hawassa, Ethiopia; Hawassa College of Health Sciences, Hawassa, Ethiopia; College of Medicine and Health Sciences, Hawassa University, Hawassa, Ethiopia; College of Medicine and Health Sciences, Hawassa University, Hawassa, Ethiopia; Sidama Regional Health Bureau, Hawassa, Ethiopia; Ethiopian Public Health Institute, Addis Ababa, Ethiopia; College of Medicine and Health Sciences, Hawassa University, Hawassa, Ethiopia

**Keywords:** Dale-Wonsho HDSS, cohort profile, birth, mortality, Ethiopia

Key Features

- The Dale-Wonsho cohort is an open population cohort established in 2017 in rural southern Ethiopia. Its primary objective is to provide a core framework for clinical trials and generate longitudinal epidemiological data that can supply up-to-date evidence and inform decision making at both regional and national levels.
- The cohort consists of 12 kebeles (a kebele is the lowest administrative unit in Ethiopia) that include all individuals permanently living in the area, with a population of 69 021 people registered as of 2017, when the study began. At the time, approximately 51.5% (35 582) of the population were male and 48.5% (33 439) were female. Women of reproductive age account for a quarter of the total population.
- At baseline, the participation rate was 100%; however, due to various factors such as migration, death or refusal to participate, we observed a dropout rate of 0.08% between the first and second rounds of data collection.
- Data on marital status, immunization, pregnancies, births, marriages, migrations and deaths were monitored and updated biannually. The project involves a multidisciplinary team, an excellent research setup and well-trained data collectors, and is affiliated with Hawassa University.
- Cohort data are available upon request, provided that a comprehensive project proposal including details about planned use of the data is submitted.

## Why was the cohort set up?

The Dale-Wonsho cohort is located in southern Ethiopia. It is one of the most densely populated areas in southern Ethiopia, with a population density of 533 persons/km^2^. The Dale district covers an area of 30 212 km^2^, situated at latitude 6.6931° or 6° 41′ 35″N and longitude 38.35461° or 38° 21′ 17″E, with a population of 268 839 people and an estimated 53 768 households.^1^^,^^2^ Dale district consists of 36 rural and two urban kebeles, which are the lowest administrative structures. Wonsho district covers an area of 14 528 km^2^, inhabited by 129 730 people living in 17 rural and one urban kebele, with a total of 21 857 households. Both districts are renowned for their coffee and crop production and sale. Dale district has 10 health centres and 33 health posts, whereas Wonsho district has five health centres and 17 health posts.^3^^,^^4^ Agriculture is the primary source of income for the inhabitants of the site, who primarily produce Enset (false banana), coffee, maize, teff, barley and a variety of fruits, vegetables and livestock. There is one government general hospital and 15 health centres that provide medical services, as well as 53 health posts and eight private clinics.

The Dale-Wonsho cohort was established in 2017 to address the lack of comprehensive health and demographic data in rural southern Ethiopia. This issue is particularly pressing in developing countries like Ethiopia, where resource constraints limit the ability to conduct regular registration, census and national population-based surveys and surveillance systems. As a result, there is often a dearth of health and demographic information at the community or population level, making it difficult to assess health trends accurately and identify areas in need of intervention.

To help address this problem, the site was designed to provide reliable demographic and health indicators for a rural population. By establishing a continuous systematic registration of vital events, it aims to complement episodic demographic and health surveys (DHS) by collecting longitudinal data over time, often with multiple household surveys. This approach allows for a more comprehensive understanding of health trends and patterns in the region, enabling researchers and policy makers to make better-informed decisions about public health and development initiatives. Overall, the cohort serves as a valuable resource for both researchers and policy makers, providing them with accurate and up-to-date data to guide decision making and improve the health and wellbeing of populations in rural Ethiopia.

## Who is in the cohort and how often have they been followed up?

**Figure 1.**
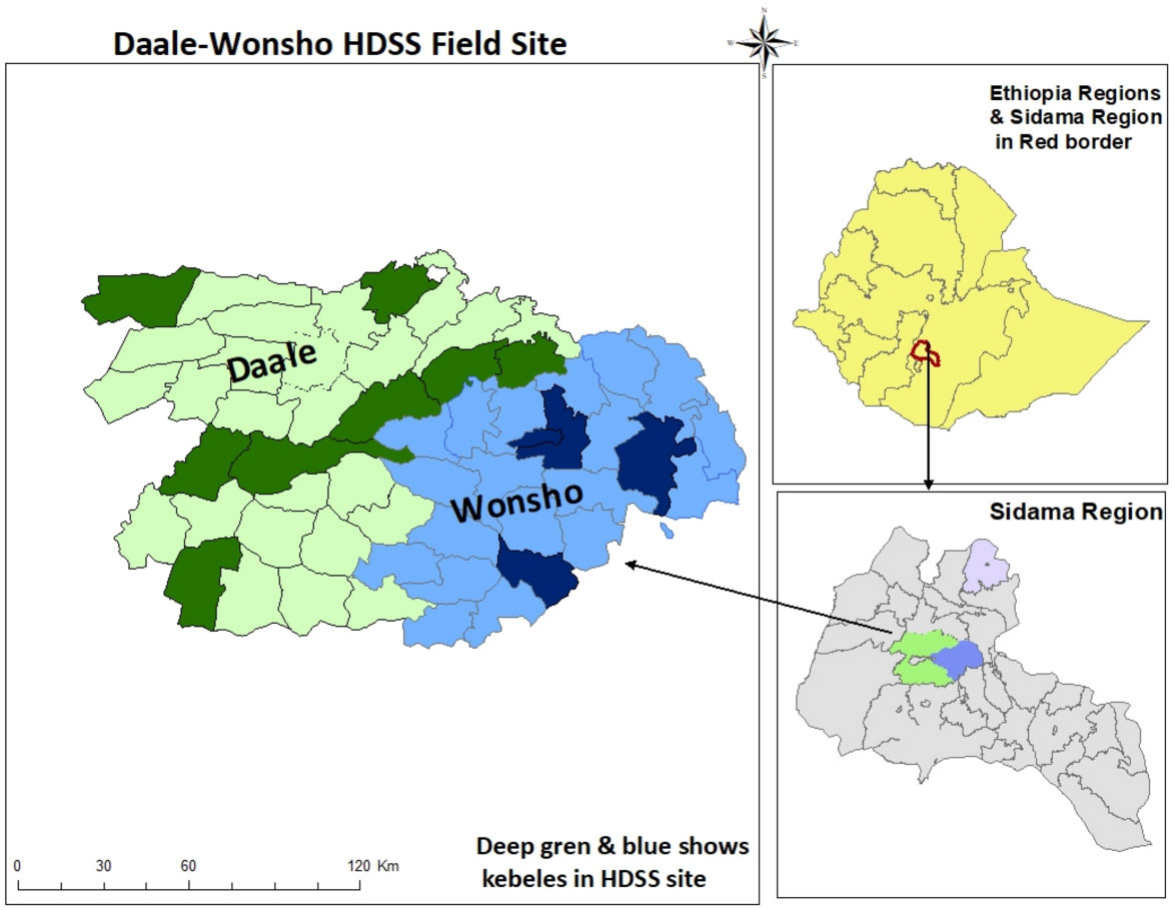
Map of Dale-Wonsho HDSS and the 12 kebeles of the study sites, Sidama region, Ethiopia

The Dale-Wonsho demographic and health survey system [HDSS] is an open cohort. We enrol new participants and update our database every 6 months, including 12 kebeles of all individuals who reside permanently in the area (Figure 1). Individuals who are visiting or have lived in the study site for less than 6 months are not considered residents. During the initiation of the HDSS in 2017, the study area was mapped, and a census of the population was conducted. The estimated number of households in the selected kebeles was 13 965, with a total population of 69 021 individuals registered. Following the baseline census, household and individual attributes have been updated biannually. All residents were assigned a unique 12-digit identification number comprising the 3-digit ‘Got’ code, 3-digit for Open HDS, 3- digit household number and a 3-digit personal number serially assigned to residents of each household. Individuals enter the cohort through initial enumeration, birth or in-migration and data collection is completed within 3 months.

As such, we have a dynamic population under observation, and our sample size changes over time. We did experience some non-participation from households within the kebeles included in our study area. Specifically, we had a participation rate of 100% at baseline, all households agreeing to participate in the first round of data collection. However, due to factors such as migration, death or refusal to participate, we observed a dropout rate of 0.08% between the first and second rounds of data collection.

## What has been measured?

The site collects longitudinal, population-based data on demographics (including births, deaths, marriages, pregnancies and migrations), health and socioeconomic information from the surveillance population. During the baseline survey in 2017, data were collected on different variables such as sociodemographic characteristics, birth, income, family planning and disease conditions. Now in the second round and third round (not completed yet), it has added variables such as detailed birth information, immunization, antenatal care follow-up, migration and marital status changes (Table 1).

**Table 1.** Information collected and its round of the Dale-Wonsho HDSS, Sidama region, Ethiopia

Variable	Information	Baseline measures	Follow-up survey measures	Additional measures
Compound and household details	Household number, individual ID number, household head, years since household was in current location, information on women of reproductive age, wealth index	X	X	
Individuals	Latitude and longitude, name, father ID, mother ID, individual ID, age, relation to household head, sex, educational status, marital status, occupation		X	
Re-census	Latitude and longitude, household number, individual ID number, household head, years since household was in current location, information on women of reproductive age, wealth index, house condition			Every 5 years
Pregnancy observation	Mother’s name, last normal menstrual period, antenatal care follow-up, number of pregnancies		X	
Pregnancy outcome	Mother’s name, father’s name, date of birth, outcome, number of births, place of delivery, vaccination status, type and date of vaccination		X	
Death	Name, date of death, cause of death, place of death		X	
In-migration	Date of in-migration, type of in-migration (within or outside study site), former residence (urban or rural), reason for in-migration, household head name, relation to household head, name, sex, date of birth of immigrant, educational status, marital status, occupation		X	
Out-migration	Date of out-migration, type of out-migration (within or outside study site), name, destination of migration), reason for out-migration		X	
Marital status	The name of the person who changed the marriage, husband’s name, previous marital status, current status		X	

In upcoming rounds, the survey will expand to include an additional district, and there are also plans to implement verbal autopsies using World Health Organization standards, as well as to collect data on other diseases. Although there is a plan to link data with other datasets, this has not yet begun.

## What has it found?

**Figure 2.**
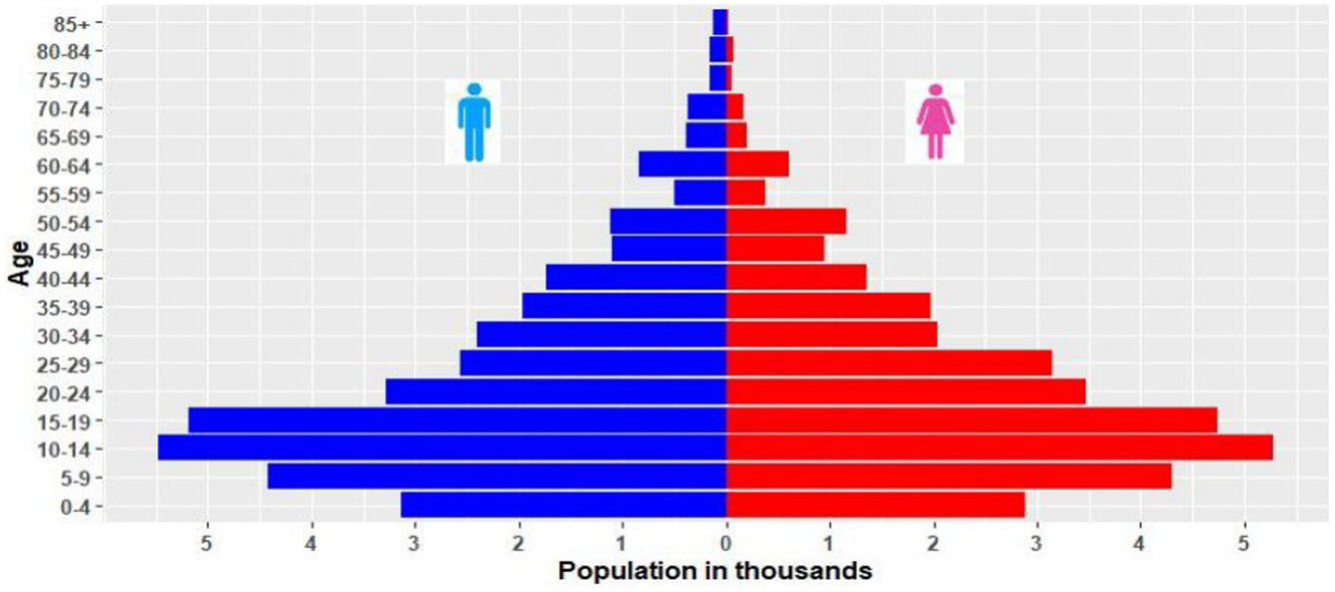
Population pyramid of Dale-Wonsho HDSS, Sidama region, Ethiopia

Table 2 shows the demographic characteristics of Dale-Wonsho HDSS. In 2017, the district had an estimated population of 69 021 and 13 965 households, with an average of 4.94 individuals per household. The population's sex ratio was 1.064 and the dependency ratio was 0.672, with a predominance of young dependency (0.63) compared with old dependency (0.042). The total dependency ratio for the Dale-Wonsho-HDSS site seems relatively lower (67.2 dependents for every 100 working-age individuals) compared with Kersa HDSS,^5^ and it is also half lower than the Ethiopian 2016 DHS report (DHS 2016). This might be due to lower total fertility rates in Dale-Wonsho HDSS. As in many developing nations, the broader base and narrow tip of the pyramid shows high fertility and lower life expectancy in the districts (Figure 2).

**Table 2.** Selected key demographic characteristics of Dale-Wonsho HDSS at baseline in 2017, Sidama Region, Ethiopia

Characteristic	Finding
Mid-year population	69 021
Total houses	13 965
Persons per household	4.94
Sex ratio (male:female)	1.064:1
Sex ratio at birth (male:female)	1.03:1
Total dependency ratio	0.672:1
Young-age dependency ratio (age <15 years)	0.63:1
Old-age dependency ratio	0.042:1
Women of reproductive age (15–49 years)	25.6%
Total number of live births	713
Crude birth rate per 1000	40.3
Crude death rate per 1000	1.3
Infant mortality rate per 1000 live births	9.82
Child mortality rate per 1000 under-five population	4.53
Under-five mortality per 1000 live births	26.4

Moreover, as shown in Table 2, most of the baseline's key findings align with reports from the Ethiopian Demographic and Health Survey 2021 (1) and other similar HDSS reports. Comparative similarities with the study reports of other HDSS site reports of Ethiopia are also observed.^5^^,^^6^ Lower total dependency rates might be due to lower total fertility rates in Dale-Wonsho HDSS (2.9).^7^ The crude death rate at our HDSS site is notably lower than both the national average and that of other HDSS sites.^5^^,^^8^ This divergence may be attributed to the under-reporting of neonatal and infant deaths within our study area. In Sidama culture, there is a belief that openly grieving the loss of a young child could potentially attract further misfortune to the family, and the death of an infant under 1 year of age is considered a delicate and taboo topic for discussion.

The crude birth rate (CBR) was 40.3 per 1000 of the population, which is higher than the findings in EDHS and Kersa HDSS. This might be due to most of the population in the Dale Wonsho HDSS residing in rural areas. CBR in rural areas is expected to be high compared with urban areas because of stronger cultural and societal preferences for larger families. So far, we have conducted two community-based studies in the area. One mixed-method study conducted in the area identified a tendency to prefer non-disclosure of diseased infants. This study also implied that measuring the accurate age of the residents demands a better approach.^9^

The Dale-Wonsho HDSS aims to use the data obtained to develop scholarly papers and policy documents. Future analyses and collaborations will focus on time trends in 2017–22 to provide a detailed evaluation of the outcomes of the pregnancy distribution and determinants of maternal and child health and survival outcomes, mortality trends, population dynamics, migration, marriages and vaccine coverage stratification by urban and rural households. In addition, assessments of fertility patterns and the effect of antenatal care on institutional delivery will be conducted. We also welcome any collaboration in these analyses or other areas.

## What are the main strengths and weaknesses?

Dale-Wonsho HDSS is supported through thematic research of Hawassa University and Ethiopian Public Health Institution (EPHI). The site has a good working relationship with the communities and other partners. The questionnaires were tested in other HDSS in Ethiopia. It is a highly functional platform for conducting randomized controlled trials; accumulated linked and detailed data allow a wide variety of retrospective, population-based studies, and the use of HRS-2 database software for data storage is also a strength. However, houses are visited biannually, and some vital events may be missed. Notably, neonatal deaths are often under-reported due to cultural taboos, and pregnancies may not be reported during their early stages for similar reasons. Hence, births that occur within 6[ months of a visit may be reported, but pregnancy reports might not be available.

## Can I get hold of the data? Where can I find out more?

Dale-Wonsho HDSS is ready to collaborate with researchers nationally and internationally, including those performing multi-site large-scale research projects. Hence, data can be shared within the framework of a collaboration agreement. A formal request can be made to the scientific committee through [tamisodebiso@hu.edu.et].

## Ethics approval

The study complies with all ethical principles and base-line and other surveys were approved (IRB-CMHS-86–17) by Hawassa University’s institutional review board.

## Data Availability

See’ Can I get hold of the data?’ above.
